# Antigen‐specific TCR‐T cells from Rag2 gene‐deleted pluripotent stem cells impede solid tumour growth in a mouse model

**DOI:** 10.1111/cpr.13389

**Published:** 2023-01-02

**Authors:** Bingyan Wu, Qi Zhang, Pingshan Hong, Lijuan Liu, Huan Peng, Chengxiang Xia, Tongjie Wang, Yao Wang, Qitong Weng, Xiaofei Liu, Yang Geng, Jinyong Wang, Hongling Wu

**Affiliations:** ^1^ CAS Key Laboratory of Regenerative Biology, Guangdong Provincial Key Laboratory of Stem Cell and Regenerative Medicine, Guangzhou Institutes of Biomedicine and Health, Chinese Academy of Sciences Guangzhou China; ^2^ University of Chinese Academy of Sciences Beijing China; ^3^ State Key Laboratory of Stem Cell and Reproductive Biology, Institute for Stem Cell and Regeneration, Institute of Zoology, Chinese Academy of Sciences Beijing China; ^4^ Liwan People's Hospital of Guangzhou Guangzhou China; ^5^ Beijing Institute for Stem Cell and Regenerative Medicine Beijing China; ^6^ Bioland Laboratory (Guangzhou Regenerative Medicine and Health Guangdong Laboratory) Guangzhou China

## Abstract

The technology of adoptive transfer of T‐cell receptor (TCR) engineered T cells is wildly investigated as it has the potential to treat solid cancers. However, the therapeutic application of TCR‐T cells is hampered by the poor quality derived mainly from patients' peripheral blood, as well as heterogeneous TCRs caused by the mismatch between transgenic and endogenous TCRs. To improve the homogeneity, antigen‐specificity and reduce possible autoreactivity, here we developed a technique to generate antigen‐specific T cells from Rag2 gene‐deleted pluripotent stem cells (PSCs) and further measured their anti‐tumour efficacy. PSCs were first targeted with OT1 TCR into the Rag2 locus to prevent TCR rearrangement during T‐cell development. The engineered PSCs were then differentiated through a two‐step strategy, in vitro generation of haematopoietic progenitor cells, and in vivo development and maturation of TCR‐T cells. Finally, the response to tumour cells was assessed in vitro and in vivo. The regenerated OT1‐iT displayed monoclonal antigen‐specific TCR expression, and phonotypic normalities in the spleen and lymph node tissues. Importantly, the OT1‐iT cells eliminated tumour cells while releasing specific cytokines in vitro. Furthermore, adoptive transfer of OT1‐iT cells suppresses solid tumour growth in tumour‐bearing animals. Our study presents a novel and straightforward strategy for producing antigen‐specific TCR‐T cells in vivo from PSCs, allowing for allogeneic transplantation and therapy of solid tumours.

## Graphical abstract

B. Wu et al. presented a novel and straightforward strategy for producing antigen‐specific TCR‐T cells in vivo from Rag2 gene‐deleted pluripotent stem cells. Those T cells showed monoclonal antigen‐specific TCR expression and effectively inhibited tumour growth in tumour‐bearing models.

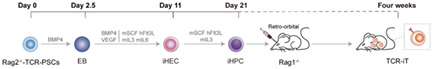

## 1 INTRODUCTION

T cells engineered to express TCRs have shown encouraging results in the therapy of melanoma and sarcoma.^1^, ^2^, ^3^, ^4^, ^5^ TCR‐T cells, unlike CAR‐T cells, can recognize particular antigens on or within of cancer cell membranes as peptides that attach to major histocompatibility complex (MHC) molecules, allowing them to recognize a far broader spectrum of antigens.^6^ However, a variety of obstacles have hindered the therapeutic application of TCR‐T cells. For instance, transgenic TCRs compete with endogenous TCRs, which leads to dilution of tumour specific TCRs and decreased T cell avidity and anti‐tumour efficacy. Furthermore, because TCRs are heterodimers, the α and β chains of transgenic TCRs can easily mismatch with α and β chains of endogenous TCRs to generate new hybrid receptors, possibly raising the risk of autoimmune disease or causing other safety issues.^7^, ^8^, ^9^, ^10^ Several strategies for reducing endogenous TCRs and improving the transgenic TCRs have been developed. The elimination of the endogenous αβ chains of TCRs is being investigated. Many research groups have explored interrupting T‐cell receptor constant alpha chain (TRAC) or beta chain (TRBC). Torikai et al. showed in 2012 that T cells with TRAC or TRBC knocked off using ZFN have strong anti‐tumour activity.^11^ With the development of gene editing technology in recent years, it is now simple to delete TRAC or TRBC using TALEN or CRISPR‐Cas9.^12^, ^13^, ^14^, ^15^ However, the methods described above are based on mature peripheral T cells which are not amenable to precise gene editing and are susceptible to integration mutations. Those approaches are also limited by the number of mature peripheral T cells. In addition, the manufacturing process of prolonged infection and in vitro expansion may result in T‐cell exhaustion, and thus reduces functionality.

Pluripotent stem cells (PSCs) provide an unlimited source of cells that can be genetically manipulated and differentiated into more specialized T cells.^16^, ^17^, ^18^ Minagawa et al. recently reported the generation of TCR‐stabilized CD8αβ T cells from T‐iPSCs via knockout of recombination activating genes 2 (Rag2).^19^ The Rag1 or Rag2 gene, which mediates the TCR V(D)J recombination during CD4 and CD8 double negative and CD4 and CD8 double positive stages, might be the ideal targets for reducing endogenous TCRs.^20^ However, the initial T‐iPSCs were obtained by laborious screening of antigen‐specific T‐cell clones and challenging reprogramming them into T‐iPSCs. The production and selection of T‐iPSCs are time‐ and money‐consuming processes.

It would be more practicable to develop an approach for directly producing transgenic TCR‐T (TgTCR‐T) cells from engineering PSCs. Previously, our groups reported a novo method to generate T cells in vivo by overexpressing particular transcription factors *Runx1* and *Hoxa9* in PSCs.^21^, ^22^ Here, we used CRISPR–Cas9 technology to further target transgenic TCR into the Rag*2* locus of PSCs. We validated that PSCs' capacity to differentiate into T cells was not impeded by genetic engineering. Importantly, we showed that this approach can effectively generate a considerable number of desired tgTCR‐T cells in vivo. These PSC‐derived tgTCR‐T cells exhibited high TCR‐mediated specificity as well as a powerful anti‐tumour activity. Our founding might provide an efficient strategy to obtain limitless and stable TCR‐T cells from PSCs that have been genetically targeted to a tumour antigen of interest.

## 2 MATERIALS AND METHODS

### 2.1 Rag2^−/−^‐OT1‐iR9‐PSCs generation

iR9‐PSCs generation has been previously described.^21^ The donor template, which included OT1 TCRα‐p2a‐OT1 TCRβ under the control of CAG promoter with internal ribosome entry site‐GFP and PKG promoter‐puromycin, was cloned and assembled to the vector flanked by Rag2 homology left and right arms (about 1000 nucleotides/each arm). Two sgRNAs for the exon 3 of Rag2 (sgRNA1: CATCAATATATCATTCACGG; sgRNA2: ATTGACGTGGTGTATAGTCG) were cloned to the Cas9 expression vector (PX330). The two Cas9–sgRNA expression vectors and the donor template were then electroporated into iR9‐PSCs together. Rag2^−/−^‐OT1‐iR9‐PSC clones (1#) with the deletion mutation on one allele and OT1 knockin on another Rag2 allele was chosen.

### 2.2 Mice

Inbred Rag1^−/−^ mice (C57BL/6 background) were obtained from Dr.Z.Liu from Institute of Biophysics (CAS). SCID (CB17/Icr‐Prkdc^scid^/IcrlcoCrl) mice and C57BL/6 (CD45.2^+^) mice were purchased from Beijing Vital River Laboratory Animal Technology. Mice were bred in the SPF‐grade animal facility of the Guangzhou Institutes of Biomedicine and Health, Chinese Academy of Sciences (GIBH, CAS). All animal experiments were conducted in accordance with the guidelines and regulations of Institutional Animal Care and Use Committee of Guangzhou Institutes of Biomedicine and Health (IACUC‐GIBH).

### 2.3 Cell culture

Mouse embryonic fibroblasts (MEFs) were isolated from 13.5 d.p.c C57BL/6 mouse embryos. MEFs were cultured in DMEM/high glucose (HyClone), 10% FBS (Natocor) supplemented with 1% non‐essential amino acids (NEAA; Gibco). PSCs were maintained on feeder layers (MEFs) in DMEM/high glucose medium supplemented with 15% FBS (Gibco), 1% NEAA (Gibco), 1% GlutaMAX (Gibco), 1% sodium pyruvate (Gibco), 0.1 mM β‐mercaptoethanol (Sigma), 1 μM PD0325901 (Selleck), 3 μM CHIR‐99021 (Selleck), and 1000 U/ml LIF (Peprotech). E.G7‐OVA cell line (ATCC) was cultured in RPMI‐1640 (Gibco) supplemented with 10% FBS (Natocor), 1% GlutaMAX (Gibco), 1% sodium pyruvate (Gibco), and 0.1 mM β‐mercaptoethanol (Sigma). B16F10 melanoma cell line was purchased from the Cell Bank of Chinese Academy of Sciences and stably transduced with chicken OVA cDNA to generate B16F10‐OVA cell line.^23^ B16F10‐OVA cells were cultured in complete medium [RPMI‐1640 (Gibco) supplemented with 10% FBS (Natocor), 1% sodium pyruvate (Gibco), and 1% GlutaMAX (Gibco)]. OP9‐DL1 cells were cultured in α‐MEM supplemented with 15% FBS and 1% GlutaMAX (Gibco). The AFT024 cell line (ATCC) was maintained in DMEM/high glucose and 10% FBS (Natocor) supplemented with 0.1 mM β‐mercaptoethanol (Sigma) and 1% sodium pyruvate (Gibco).

### 2.4 Rag2^−/−^‐OT1‐iT cells differentiated from Rag2^−/−^‐OT1‐iR9‐PSCs

We used a stepwise protocol to differentiate Rag2^−/−^‐OT1‐iR9‐PSCs into Rag2^−/−^‐OT1‐iT cells, as previously described.^21^ Briefly, for EB formation, Rag2^−/−^‐OT1‐iR9‐PSCs were dissociated, resuspended at 100,000 cells/ml and inverted at 20 μl/drop in 15 cm dishes in BDM medium [BDM: IMDM, 15% FBS (Gibco), 200 μg/ml iron‐saturated transferrin (Sigma), 0.1 mM β‐mercaptoethanol (Sigma), 1% GlutaMAX, and 50 μg/ml ascorbic acid (Sigma)] with 5 ng/ml BMP4 (Peprotech). On Day 2.5, the EBs were then transferred to the gelatinized plates in BDM with 5 ng/ml BMP4 and 5 ng/ml VEGF (PeproTech)]. On Day 6, the medium was changed to BDM with doxycycline (1 μg/ml; Sigma) and a cocktail of 2% conditioned medium produced from the supernatants of AFT024‐mSCF, AFT024‐hFlt3L, AFT024‐mIL3, and AFT024‐mIL6 cell culture. On Day 11, iHECs (CD31^+^CD41^low^CD45^−^c‐kit^+^CD201^+^, 1000–5000 iHECs/well, 12‐well plate) were sorted and transferred to OP9‐DL1 monolayer in EM medium (α‐MEM with 15% DFBS (HyClone), 200 μg/ml iron‐saturated transferrin, 0.1 mM β‐mercaptoethanol, 1% GlutaMAX, 50 μg/ml ascorbic acid, 2% conditioned medium derived from supernatants of AFT024‐mSCF, AFT024‐hFlt3L, and AFT024‐mIL3 cell culture and 1 μg/ml doxycycline). On Day 21, iHPCs were collected and transplanted (3.0 × 10^6^ cells/recipient mice) into sublethally irradiated Rag1^−/−^ recipients (8–10 weeks, 3.5 Gy) by retro‐orbital veins. Dox (1.0 mg/ml) was supplied in the co‐trimoxazole‐containing drinking water (Tianjin Lisheng Pharmaceutical Co., Ltd.) to assure the expression of exogenous *Runx1* and *Hoxa9* for 2 weeks. Around 4 weeks later, Rag2^−/−^‐OT1‐iT cells from Rag1^−/−^ mice were detected by flow cytometry. Rag2^−/−^‐OT1‐iT cells from the spleens of Rag1^−/−^ mice transplanted with iHPCs were then harvested and used for in vitro assays or adoptive transfer experiments.

### 2.5 Flow cytometry and cell sorting

All cells were preincubated with CD16/32 Fc blocking antibody before staining related antibodies to eliminate nonspecific binding of antibodies to FcγR. Flow cytometry was performed on a LSRFortessa (BD) or LSRFortessaTMX‐20 (BD), and cell sorting was done on a FACSAriaTM III (BD). The flow data were analysed with FlowJo (Three Star). In this study, the monoclonal antibodies of CD2 (RM2‐5), CD3 (145‐2C11), CD4 (GK1.5), CD8a (53‐6.7), CD19 (eBio1D3), CD31 (390), CD41 (eBioMWReg30), CD45 (30‐F11), c‐Kit (2B8), CD201 (eBio 1560), CD11b (M1/70), B220 (RA3‐6B2), Gr1 (RB6‐8C5), NK1.1 (RK136), CD11c (N418), Ter119 (TER‐119), Sca‐1 (D7) CD44 (IM7), CD62L (MEL‐14), CD69 (H1.2F3), CD25 (PC61.5) TCR Vα2 (B20.1), TCR Vβ5 (MR9‐4), and Streptavidin‐PE‐Cy7 were brought from eBioscience or BioLegend. Rag2^−/−^‐OT1‐iR9‐PSCs were stained intracellularly with eBioscience Intracellular Fixation and Permeabilization Buffer Set.

### 2.6 Rag2^−/−^‐OT1‐iT cells killing assay in vitro

The release of lactate dehydrogenase was measured using the Non‐Radioactive Cytotoxicity Assay kit (Promega). To generate effector T cells, Rag2^−/−^‐OT1‐iT cells from the spleens of Rag1^−/−^ recipients, WT‐T cells from the spleens of C57BL/6 mice, and OT1‐T cells from the spleens of OT1 mice were first enriched using MicroBeads (Miltenyi Biotec) by depleting the Ter119^+^CD19^+^CD11b^+^Gr1^+^B220^+^NK1.1^+^CD11c^+^ cells, and then activated for 3 days using the Dynabeads Mouse T‐Activator CD3/CD28 (Gibco) in T‐cell medium [RPMI‐1640 with 100 IU/ml rhIL‐2 (PeproTech), 2 ng/ml mIL‐7 (PeproTech), 1% GlutaMAX (Gibco), 1% NEAA (Gibco), 0.1 mM β‐mercaptoethanol, and 10% FBS (Ausbian). The effector T cells of Rag2^−/−^‐OT1‐iT were incubated with the target cells of E.G7‐OVA (1.0 × 10^4^ cells/well, 96‐well plate) in phenol‐free RPMI‐1640 medium with 2% FBS (Ausbian) (100 μl/well). The WT effector T cells incubated with E.G7‐OVA were used as the negative controls, while the OT1 effector T cells incubated with E.G7‐OVA were used as the positive controls. The effector T cells were added at the various effector:target (E:T) ratios of 1:1, 2:1, 5:1, or 10:1. Following a 48‐h incubation, 2.5 μl of each supernatant was collected and analysed T‐cell cytotoxicity according to the instructions of the Non‐Radioactive Cytotoxicity Assay kit (Promega). To evaluate cytokine production, 50 μl of each supernatant from the cytotoxicity assay after 72‐h incubation were collected and analysed according to the instructions of IFN‐γ (R&D Systems) and TNF‐α ELISA detection kits (BioLegend).

### 2.7 Anti‐tumour assay of Rag2^−/−^‐OT1‐iT cells and Rag2^−/−^‐OT1‐iHPCs in vivo

To study the anti‐tumour effect of Rag2^−/−^‐OT1‐iT cells in vivo, the SCID mice were subcutaneously transplanted with B16F10‐OVA cells (1.5 × 10^5^ cells/mice) on Day 0. Rag2^−/−^‐OT1‐iT isolated from the spleens of Rag1^−/−^ recipients 6 weeks after transplantation, and OT1‐T cells isolated from the spleens of OT1 mice were expanded for 7 days using the Dynabeads Mouse T‐Activator CD3/CD28 (Gibco) in above mentioned T‐cell medium. Then the effector T cells of Rag2^−/−^‐OT1‐iT and OT1‐T (1.0 × 10^7^ cells/mice) were retro‐orbitally inoculated to the irradiated tumour‐bearing CB‐17 SCID mice (3.5 Gy) on Day 12, respectively. As negative controls, PBS was retro‐orbitally inoculated to the irradiated tumour‐bearing SCID mice. To study the anti‐tumour effect of Rag2^−/−^‐OT1‐iHPCs in vivo, Rag2^−/−^‐OT1‐iHPCs (3.0 × 10^6^ cells/mice) were retro‐orbitally inoculated to the irradiated tumour‐bearing Rag1^−/−^ mice (3.5 Gy) subcutaneously transplanted with B16F10‐OVA cells (1.0 × 10^5^ cells/mice) prior to 3 days. PBS was retro‐orbitally inoculated to the irradiated B16F10‐OVA tumour‐bearing Rag1^−/−^ mice as negative controls. Tumour size (length×width) was measured every 2 days. Mice suffered from heavy tumour burdens (tumour size larger than 400 mm^2^) were euthanized for ethical consideration. The survival time of tumour mice was recorded.

### 2.8 Statistics

All data presented in the figure panels were performed using GraphPad Prism and based on at least three replicated samples. Two‐tailed Student's *t*‐test, one‐way ANOVA, and log‐rank tests were used by SPSS software to calculate statistical significance. *p* > 0.05 was defined as no significance (NS); *p < 0.05; **p < 0.01; ***p < 0.001.

## 3 RESULTS

### 3.1 Targeted integration of a TCR into Rag2 locus of PSCs

**FIGURE 1:**
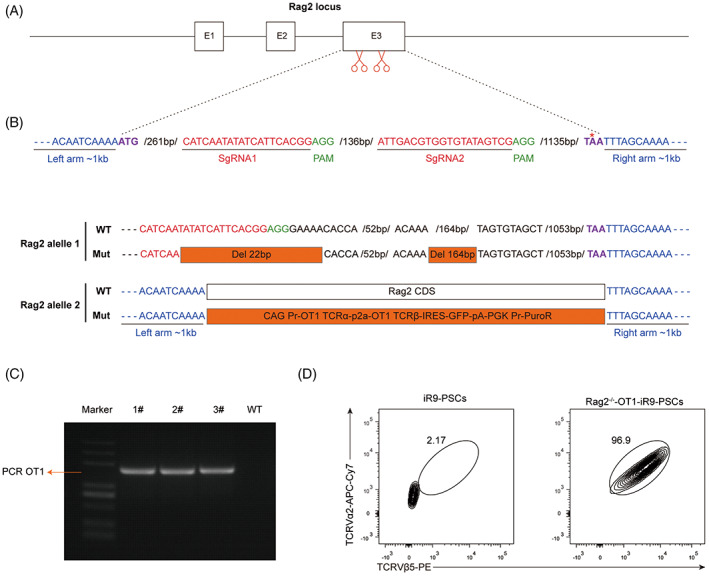
Generation of Rag2^−/−^‐OT1‐iR9‐PSCs. (A) Diagram of sgRNA targeting the Rag2 locus. Two sgRNA sequences, sgRNA1 and sgRNA2, are marked in red, whereas the PAM sequences are showed in green. The promotor and the stop code are highlighted in purple. The blue letters indicated the left and right homologous arms. (B) Sanger sequencing diagram of Rag2^−/−^‐OT1‐iR9‐PSC clone (1#). The WT and the Mut sequence of Rag2 (one allele deleted; the other allele inserted) are presented. (C) OT1 PCR detection of Rag2^−/−^‐OT1‐iR9‐PSC clones. 1#, 2#, 3# represent different clones of Rag2^−/−^‐OT1‐iR9‐PSCs; WT represents iR9‐PSCs. (D) Expression of OT1 in Rag2^−/−^‐OT1‐iR9‐PSC clone (1#) was detected by intracellular staining using anti‐mouse Vα2 and Vβ5 antibody. iR9‐PSCs were used as negative control.

Our previous studies found that ectopic expression of *Runx1* and *Hoxa9* in mouse PSCs (iR9‐PSCs) efficiently induced the differentiation of haematopoietic progenitor cells (iHPCs) in vitro, and then successfully generated mature T lymphocytes in vivo.^21^, ^22^ To further produce tumour antigen‐specific CD8 T cells, we selected OVA‐antigen TCR (OT1, MHC‐I restricted, recognizing OVA_257‐269_), a well‐characterized CD8 TCR. To prevent the production and rearrangement of endogenous TCRs, we used the CRISPR–cas9 technology to knockout recombination activating genes 2 (Rag2) and knockin OT1 under the control of CAG promotor of iR9‐PSCs (referred to as Rag2^−/−^‐OT1‐iR9‐PSCs) (Figure 1A). According to Rag2 gene sequencing, one allele of the gene had 186 base pair deletions, while the other allele had an OT1 exchange (Figure 1B). The 186 bp deletion on one allele created a mutant protein with 62 amino acids shortened, which is crucial for the structure and catalytic activity of the Rag complex, since the minimal core of Rag2 has been considered to be residues 1‐351 of 527.^20^, ^24^, ^25^ PCR amplification and gel electrophoresis were used to validate the OT1 exchange on another allele (Figure 1C). The intracellular costaining of TCR Vα2 and Vβ5 in Rag2^−/−^‐OT1‐iR9‐PSCs was then carried to identify OT1 expression. As shown in Figure 1D, Rag2^−/−^‐OT1‐iR9‐PSCs demonstrated a high level of OT1 expression.

### 3.2 Rag2^−/−^‐OT1‐iR9‐PSCs can successfully generate desired monoclonal TCR‐T cells

**FIGURE 2:**
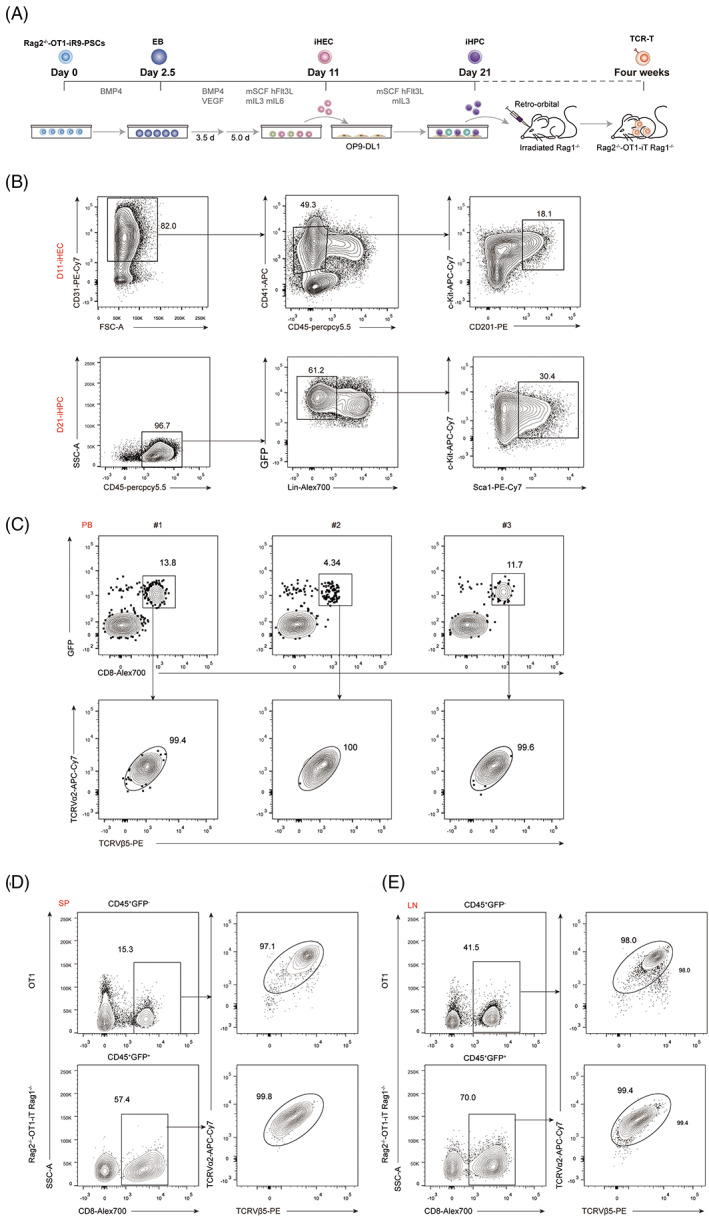
Rag2^−/−^‐OT1‐iT cells differentiation from Rag2^−/−^‐OT1‐iR9‐PSCs. (A) In vitro and in vivo differentiation protocol. Rag2^−/−^‐OT1‐iR9‐PSCs were differentiated into iHECs by EB formation for 2.5 days. The EBs were then transferred to gelatinized plates for another 3.5 days (BDM medium with BMP4 and VEGF). On Day 6, exogenous *Runx1* and *Hoxa9* were conditionally expressed in the presence of doxycycline along with haematopoietic cytokines (mSCF/mIL3/mIL6/hFlt3L) for iHEC induction. On Day 11, iHECs were sorted and aggregated with the mouse bone marrow stromal cell line OP9‐DL1 for iHPC maturation in differentiation medium (containing mSCF/mIL3/hFlt3L). On Day 21, iHPCs were transplanted into sublethally irradiated (3.5 Gy) Rag1^−/−^ mice to reconstitute T lymphopoiesis. (B) Flow cytometric analysis of iHECs on Day 11 and iHPCs on Day 21. iHECs were defined as CD31^+^CD41^low^CD45^−^c‐kit^+^CD201^+^. iHPCs were defined as Lin^−^CD45^+^GFP^+^c‐kit^+^Sca‐1^+^. All the lineage‐negative (Lin^−^) was defined as CD2^−^CD3^−^CD4^−^CD8^−^CD11b^−^Gr1^−^Ter119^−^CD19^−^NK1.1^−^TCRγδ^−^. (C) Rag2^−/−^‐OT1‐iR9‐PSCs derived mature iT cells in peripheral blood (PB) of Rag1^−/−^ recipients were analysed by flow cytometry 4 weeks after transplantation. Three million Rag2^−/−^‐OT1‐iHPCs were transplanted into each sublethally irradiated Rag1^−/−^ mouse. Data from three representative mice are shown. (D) Mature iT cells in the spleen and (E) lymph node of Rag2^−/−^‐OT1‐iT Rag1^−/−^ mouse. Representative Rag1^−/−^ mouse was transplanted with 3.0 × 10^6^ iHPCs collected on Day 21. Mature iT cells from spleen cells or lymph node cells of Rag2^−/−^‐OT1‐iT Rag1^−/−^ mouse 9 weeks after transplantation were analysed by flow cytometry. Transgenic OT1 mice were used as positive control. SP, spleen; LN, lymph nodes. Flow plots from one representative mouse of each group are shown.

We followed the same stepwise strategy for in vitro haematopoietic progenitor cells (iHPC) differentiation and in vivo transplantation of iHPCs to reconstitute T lymphopoiesis, as previously described (Figure 2A). To generate Rag2^−/−^‐OT1‐iHPCs in vitro, we first induced Rag2^−/−^‐OT1‐iR9‐PSCs differentiation as embryoid bodies for 2.5 days and then transferred them to gelatinized plates for iHECs induction for another 8.5 days. On Day 11, iHECs (CD31^+^CD41^low^CD45^−^c‐kit^+^CD201^+^) were sorted (Figure 2B) and implanted on stromal cell OP9‐DL1^26^ for iHPC maturation. On Day 21, 3.0 × 10^6^ Rag2^−/−^‐OT1‐iHPCs were transferred into Rag1^−/−^ mice that had been sublethally irradiated (3.5 Gy) (Figure 2B). Four weeks after transplantation, as expected, Rag2^−/−^‐OT1‐iR9‐PSCs developed into higher CD8^+^ Rag2^−/−^‐OT1‐iT cells in the peripheral blood (Figure 2C). Furthermore, 9 weeks after transplanted with iHPCs, CD8^+^ Rag2^−/−^‐OT1‐iT cells were seen in the spleen and lymph node of Rag1^−/−^ mice as the OT1‐T cells from transgenic mice (Figure 2D,E). Therefore, antigen‐specific TCR‐T cells can be effectively produced by PSCs genetically engineered with TCR.

### 3.3 Rag2^−/−^‐OT1‐iT cells are functional in vitro

**FIGURE 3:**
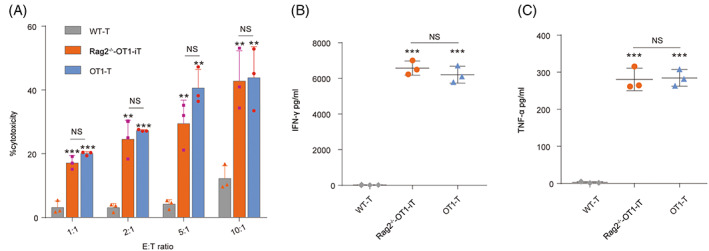
Cytotoxicity of Rag2^−/−^‐OT1‐iT cells against E.G7‐OVA cells in vitro. (A) E.G7‐OVA cells (1.0 × 10^4^/well, target cells) were incubated with Rag2^−/−^‐OT1‐iT cells, OT1‐T cells or WT‐T cells at the indicated ratios (1:1, 2:1, 5:1, or 10:1) in 96‐well plate. After a 48‐h incubation, the cytotoxic activity of T cells against E.G7‐OVA cells was detected following the instruction of the Non‐Radioactive Cytotoxicity Assay kit (Promega) (*n* = 3 per group). Results are representative of three‐independent experiments. The cytokine release of IFN‐γ (B) and TNF‐α (C) was assayed in the supernatants of 72 h co‐culture of the effector cells with the target cells at an E:T ratio of 10:1 by IFN‐γ and TNF‐α ELISA Kits according to the manufacturer's directions. *p* > 0.05 was defined as no significance (NS); *p < 0.05; **p < 0.01; ***p < 0.001.

To see if Rag2^−/−^‐OT1‐iT cells could efficiently kill tumour cells in vitro, we assessed their capacity to eradicate E.G7‐OVA tumour cells according to the prior research.^23^ Rag2^−/−^‐OT1‐iT cells were harvested from the spleen of Rag1^−/−^ mice, which were transplanted with iHPCs derived from Rag2^−/−^‐OT1‐iR9‐PSCs. Rag2^−/−^‐OT1‐iT cells were co‐incubated with the target E.G7‐OVA tumour cells in 96‐well plates (1.0 × 10^4^/well) at various E:T ratios (1:1, 2:1, 5:1, or 10:1), whereas OT1‐T cells from OT1 transgenic mice were used as a positive control. After a 48‐h incubation, Rag2^−/−^‐OT1‐iT cells exerted superior killing of E.G7‐OVA cells than WT‐T cells, and they were as effective as OT1‐T cells (Figure 3A). Furthermore, specific cytokine releases of IFN‐γ and TNF‐α were higher in the Rag2^−/−^‐OT1‐iT cell and OT1‐T cell groups compared to the WT‐T‐cell group (Figure 3B,C).

### 3.4 Rag2^−/−^‐OT1‐iT cells inhibited the growth of specific solid tumour in vivo

**FIGURE 4:**
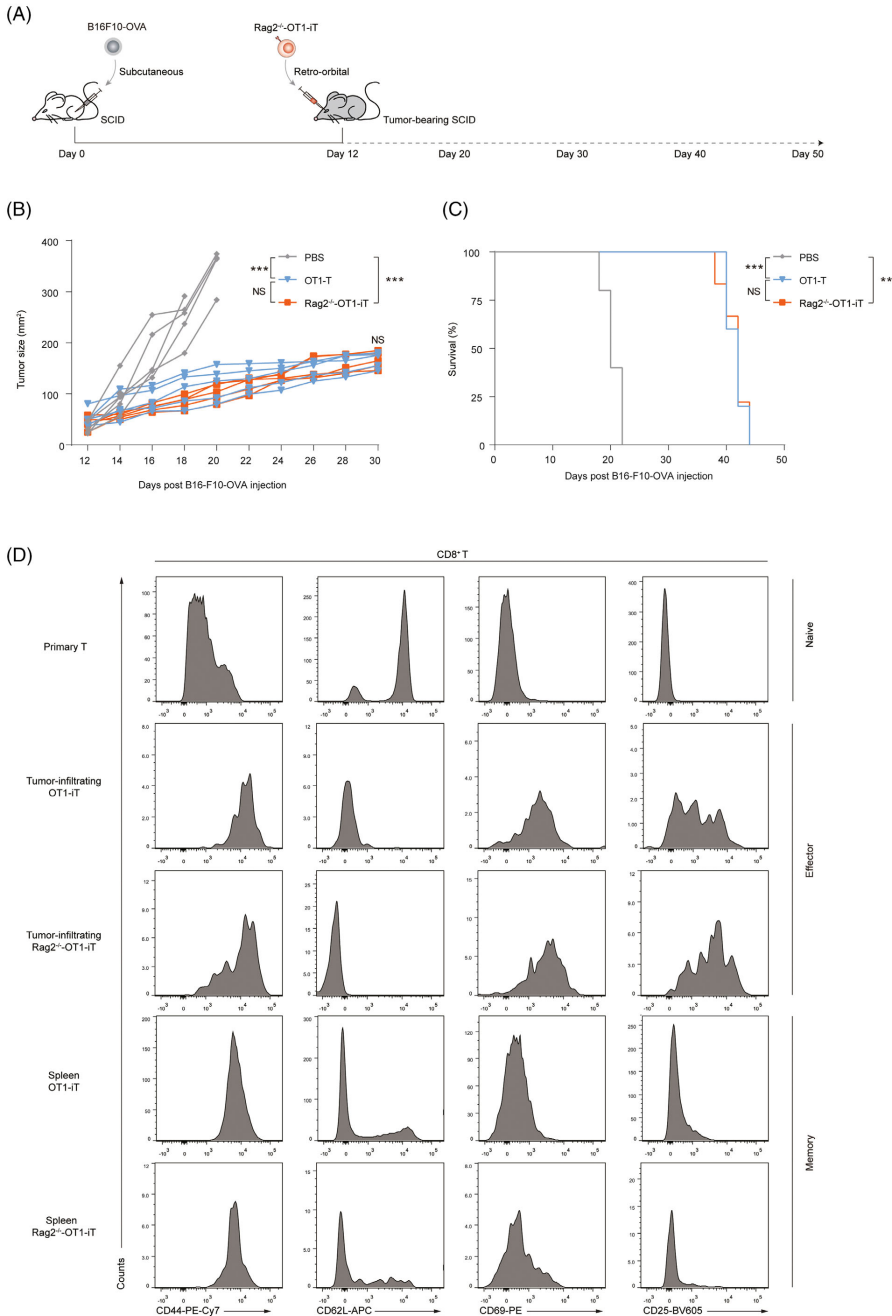
Anti‐tumour effect of adoptive transfer of Rag2^−/−^‐OT1‐iT cells in tumour‐bearing mice. (A) In vivo experimental design of Rag2^−/−^‐OT1‐iT cells anti‐tumour activity. B16F10‐OVA tumour cells (1.5 × 10^5^/mice) were subcutaneously injected into SCID mice and allowed to establish tumour‐bearing mice for 12 days. The mice were randomly divided to three experimental groups. Each group were adoptively transferred with 1 × 10^7^ Rag2^−/−^‐OT1‐iT cells from splenic Rag2^−/−^‐OT1‐iT reconstituted Rag1^−/−^ mice, 1 × 10^7^ OT1‐T cells from OT1 mice or PBS separately. The tumour size (B) and survival time (C) of each treatment group (*n* = 5 per group) were recorded. The statistical difference of tumour size on Day 18 and Day 30 was calculated. Survival analyses was performed according to the Kaplan–Meier assays, and the statistical difference was calculated by the log‐rank tests corrected with Bonferroni's method. *p* > 0.05 was defined as no significance (NS); *p < 0.05; **p < 0.01; ***p < 0.001. (D) Tumour‐infiltrating T cells were isolated by Ficoll and analysed using flow cytometry and phenotype were assigned according to CD44, CD62L, CD69 and CD25 expression as follow: naïve cells (CD44^low^ CD62L^high^ CD69^−^ CD25^−^), effector cells (CD44^high^ CD62L^low^ CD69^+^ CD25^+^), memory cells (CD44^high^ CD62L^high^ CD69^−^ CD25^−^).

To explore the therapeutic potential of these PSC‐derived OT1‐iT cells in vivo, we used a B16F10‐OVA tumour model in SCID mice. In this model, 1.5 × 10^5^ B16F10‐OVA tumour cells were subcutaneously injected into SCID mice to produce tumour growth. On Day 12, Rag2^−/−^‐OT1‐iT cells (1.0 × 10^7^/mouse) which were derived from the spleens of Rag1^−/−^ recipients transplanted with day21‐iHPCs for 6 weeks, were retro‐orbitally to these tumour‐bearing mice (Figure 4A). Every 2 days, tumour size was monitored by measuring the length and width. On Day 18 after B16F10‐OVA injection, the tumour sizes of Rag2^−/−^‐OT1‐iT‐treated mice were less than 100 mm^2^, but those of PBS‐treated tumour‐bearing mice were over 200 mm^2^ (*n* = 5, *p* < 0.001) (Figure 4B), indicating that Rag2^−/−^‐OT1‐iT cell therapy inhibits tumour growth significantly. We also observed that Rag2^−/−^‐OT1‐iT cell treatment dramatically improved tumour‐bearing mice survival as compared to PBS‐treated animals (Figure 4C). The phenotype of the infiltrating T cells in the tumour and spleen was subsequently determined. As shown in Figure 4D, primary T cells displayed the naive CD8 T cell phenotype: CD44^low^ CD62L^high^ CD69^−^ CD25^−^. After tumour stimulation in vivo, these tumour‐infiltrating Rag2^−/−^‐OT1‐iT cells exhibited the typical effector cytotoxic T cells phenotype: CD44^high^ CD62L^low^ CD69^+^ CD25^+^, whereas the splenic Rag2^−/−^‐OT1‐iT cells showed the memory phenotype (CD44^high^ CD62L^high^ CD69^−^ CD25^−^). Simultaneously, to evaluate the efficacy of PSC‐derived Rag2^−/−^‐OT1‐iT cells and the physiological OT1‐T cells from OT1 transgenic mice, we performed a detailed comparison between those two cells. Surprisingly, Rag2^−/−^‐OT1‐iT cells had a similar anti‐tumour response and phenotypic status to OT1‐T cells (Figure 4B–D). Overall, the findings indicate that iT cells regenerated from TCR‐engineered PSCs have excellent anti‐tumour activity against solid tumours due to better tumour infiltration and memory capacity.

### 3.5 Rag2^−/−^‐OT1‐ iHPCs reconstituted anti‐tumour T immunity in vivo

**FIGURE 5:**
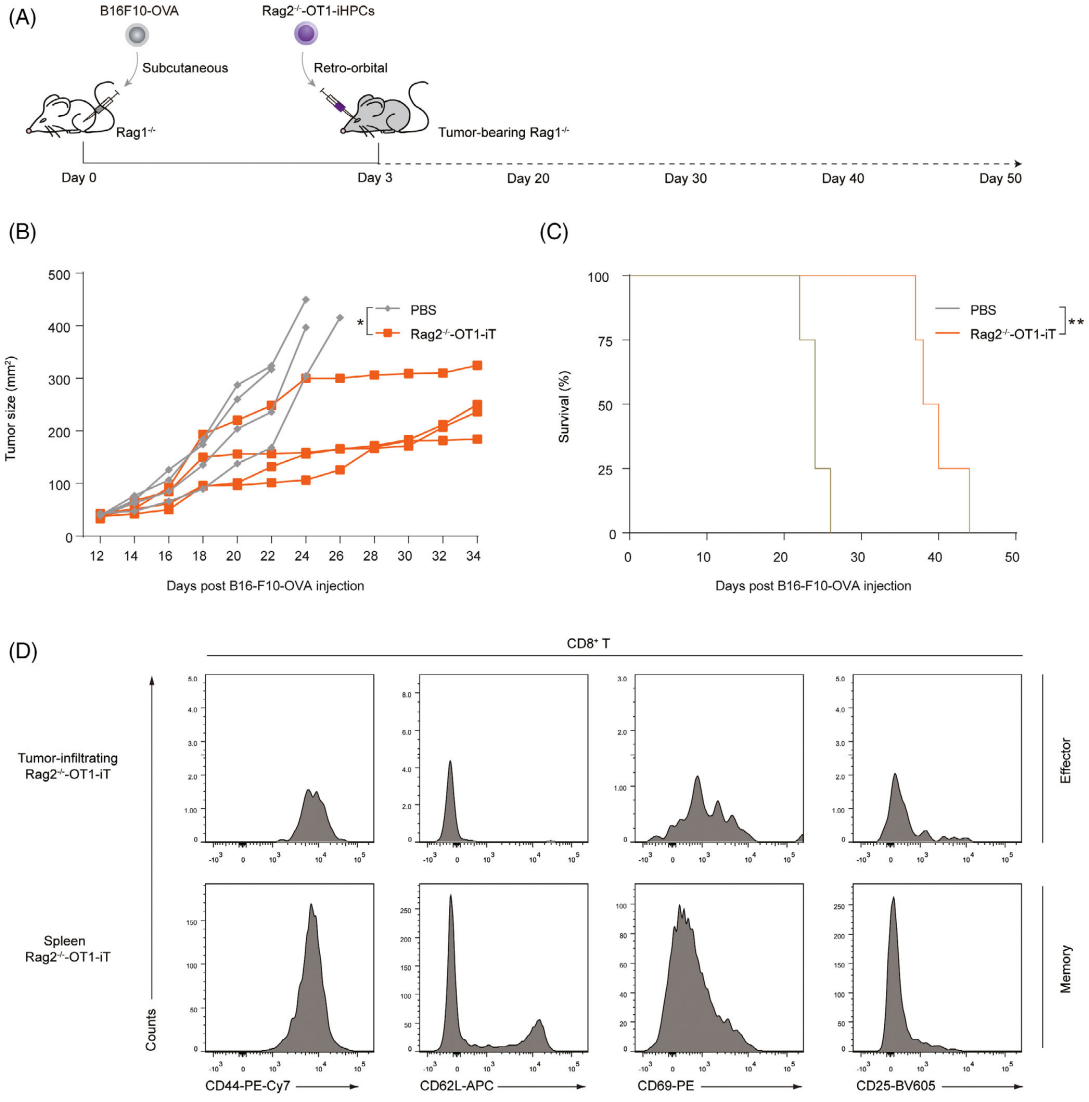
Therapeutic effect of Rag2^−/−^‐OT1‐iHPCs in tumour‐bearing mice. (A) In vivo experimental design of Rag2^−/−^‐OT1‐iHPCs anti‐tumour activity. On Day 0, Rag1^−/−^ mice were subcutaneously administered B16F10‐OVA cells. The mice were randomly divided to two experimental groups. On Day 3, each group was adoptively transferred with 3.0 × 10^6^ Rag2^−/−^‐OT1‐iHPCs from Rag2^−/−^‐OT1‐PSCs in vitro or PBS individually. The tumour size (B) and survival time (C) of each therapy group (*n* = 4) were recorded. On Day 24, the statistical difference in tumour size was calculated. Survival analysis was performed according to the Kaplan–Meier assays, and the statistical difference was calculated by the log‐rank tests, *p* > 0.05 was defined as no significance; *p < 0.05; **p < 0.01; ***p < 0.001. (D) Tumour‐infiltrating T cells were isolated by Ficoll and analysed using flow cytometry and phenotype were assigned according to CD44, CD62L, CD69 and CD25 expression as follow: naïve cells (CD44^low^ CD62L^high^ CD69^−^ CD25^−^), effector cells (CD44^high^ CD62L^low^ CD69^+^ CD25^+^), memory cells (CD44^high^ CD62L^high^ CD69^−^ CD25^−^).

We further investigated the possibility of the anti‐tumour effect from direct inoculation of Rag2^−/−^‐OT1‐iHPCs. First, tumour‐bearing Rag1^−/−^ mice were first established by engrafted with 1.0 × 10^5^ B16F10‐OVA cells via subcutaneous injection. Three days later, each recipient was irradiated and retro‐orbitally injected with 3.0 × 10^6^ Rag2^−/−^‐OT1‐iHPCs collected from Rag2^−/−^‐OT1‐iR9‐PSCs differentiated in vitro on Day 21 (Figure 5A). Three weeks later, the B16F10‐OVA tumour growth was surprisingly slower in the iHPC‐treated group than in the PBS‐treated control group, indicating that iHPCs successfully gave rise to functional OT1‐iT lymphocytes to inhibit tumour growth (*n* = 4, *p* < 0.05) (Figure 5B). iHPC‐treated mice lived noticeably longer than the PBS‐treated control group (Figure 5C). Consistent with the earlier findings, Rag2^−/−^‐OT1‐iHPC‐treated mice had infiltrating effector T cells (CD44^high^ CD62L^low^ CD69^+^ CD25^+^) in the tumour and memory T cells (CD44^high^ CD62L^high^ CD69^−^ CD25^−^) in the spleen (Figure 5D). Collectively, these results reveal that Rag2^−/−^‐OT1‐iHPCs may reconstitute the T lymphopoiesis and induce strong anti‐tumour effects.

## 4 DISCUSSION AND CONCLUSION

TCR‐T clinical studies have yield impressive outcomes. However, the supply of therapeutic T cells is a major bottleneck. TCR‐T cells generated from PSCs might be employed to address the issue. Many research groups have struggled to generate antigen‐specific CD8αβ T cells from T‐iPSCs,^17^, ^18^, ^27^, ^28^, ^29^ but here we developed a straightforward strategy for producing TCR‐iT cells from PSCs by CRISPR‐based TCR knockin to the Rag2 locus. Due to the absence of TCR rearrangement during PSCs differentiation, the regenerated TCR‐iT cells were pure and superior at antigen recognition. Similar to TCR‐T cells from transgenic mice, these TCR‐expressing PSC‐derived iT cells inhibit tumour growth in a xenograft model (Figure 4). In addition, our findings reveal that TCR‐iT cells produced from PSCs present not only an effector phenotype in the tumour tissue, but also a memory phenotype in the spleen (Figure 4), which is important for immunological surveillance. More interestingly, direct transplantation of iHPCs derived from Rag2^−/−^‐OT1‐iR9‐PSCs may also effectively suppress the development of tumours and significantly prolong the survival time of tumour‐bearing mice, indicating that iHPCs can swiftly give rise to iT cells in vivo (Figure 5). Our findings showed that either TCR‐iT cells or iHPCs can efficiently inhibit tumour growth in tumour‐bearing mouse model. TCR‐iT cells can eliminate tumours more quickly than iHPCs since they are more mature than iHPCs. In addition, TCR‐iT cells may be safer than iHPCs, because of their homogeneity and shorter lifespan, which may reduce the risk of generating unknown pluripotency cells with tumour potential. However, iHPCs may be better suited for early tumour suppression, and their clinical application requires more research.

Although the potential of PSC‐derived TCR‐T in cancer therapy has been acknowledged, how to avoid its allogeneic rejection is still a technical challenge. Graft versus host disease (GvHD) is mainly caused by graft T cell recognition of host peptide‐HLA complexed through TCR.^30^ To eliminate the TCR‐mediated GvHD, the main method is to disrupt the αβ chain of TCRs by targeting TRAC or TRBC. Because TCRs are essential for T‐cell development in thymus, such approaches can only be used on mature T cells. Here, we described a simple method for obtaining desired TCR‐iT cells from PSCs using Rag2 site‐specific recombinant. We validated that allogeneic iT cells engineered with TCR sequence in Rag2 locus have normal T‐cell development, maturation or migration since the TCR‐iT cells show normal tissue distribution and adaptive immunity as OT1‐T cell from transgenic mice. Furthermore, the TCR‐iT cells resulted in no GvHD after transplanting Rag2^−/−^‐OT1‐iR9‐iT cells (H‐2^b^) to allogenic SCID mice (H‐2^d^). Our research established a method to produce TCR‐iT cells from mouse PSCs, paving the way for human TCR‐T‐cell production. However, there are still certain issues that need to be solved for human TCR‐T‐cell regeneration. For example, more study is required to confirm whether the synergistic expression of human RUNX1 and HOXA9 can efficiently induce the differentiation of human PSCs to regenerate human T cells. Furthermore, it is necessary to perform this approach in the humanized animal model transplanted with human foetal thymus during the development of T cells.

In our study, we combined PSC with TCR engineering technologies to offer a potential new supply of ‘off‐the‐shelf’ TCR‐T cells. By targeting disruption of B2M or HLA genes, PSC‐derived TCR‐T can be further modified to reduce host versus graft disease.^31^, ^32^ If successful, these strategies may allow allogeneic transplantation of PSC‐producing TCR‐T cells.

## AUTHOR CONTRIBUTIONS

Jinyong Wang and Hongling Wu conceived and supervised the study. Bingyan Wu and Qi Zhang designed and performed the experiments, acquired and analysed the data. Pingshan Hong, Lijuan Liu, Huan Peng, Chengxiang Xia, Tongjie Wang, Yao Wang, Qitong Weng, Xiaofei Liu and Yang Geng participated in multiple experiments. Hongling Wu and Qi Zhang wrote the manuscript. Jinyong Wang and Hongling Wu reviewed, edited and finalized the manuscript. All authors approved the manuscript in its final form.

## FUNDING INFORMATION

This work was supported by the National Key R&D Program of China (2021YFA1100701, 2020YFA0112404, 2019YFA0110203), the Strategic Priority Research Program of the Chinese Academy of Sciences (XDA16010601), the Key R&D Program of Guangdong Province (2020B1111470001), the National Natural Science Foundation of China (81925002, 32100904), the Frontier Science Research Program of the CAS (QYZDB‐SSW‐SMC057) and the Science and Technology Planning Project of Guangdong Province, China (2020B1212060052).

## CONFLICT OF INTEREST

The authors declare no conflict of interest.

## Data Availability

The data that support the findings of this study are available from the corresponding author upon request.
